# Risk of Vaccine Breakthrough SARS-CoV-2 Infection and Associated Factors in Healthcare Workers of Trieste Teaching Hospitals (North-Eastern Italy)

**DOI:** 10.3390/v14020336

**Published:** 2022-02-07

**Authors:** Paolo Basso, Corrado Negro, Luca Cegolon, Francesca Larese Filon

**Affiliations:** 1 Department of Medical, Surgical & Health Sciences, University of Trieste, 34129 Trieste, Italy; paolo.basso@studenti.units.it (P.B.); negro@units.it (C.N.); larese@units.it (F.L.F.); 2 Clinical Unit of Occupational Medicine, University of Trieste, 34129 Trieste, Italy; 3 Public Health Department, University Health Agency Giuliano-Isontina (ASUGI), 34128 Trieste, Italy

**Keywords:** SARS-CoV-2, COVID-19, occupational biological risk, vaccine, healthcare workers, breakthrough infection

## Abstract

**Background:** Healthcare workers (HCWs) are particularly exposed to biological risk, including SARS-CoV-2 infection. In order to contrast the current pandemic and alleviate the burden of the disease on the healthcare system, a mass vaccination campaign against COVID-19 has been launched worldwide. **Aim** To evaluate the impact of COVID-19 vaccination in HCWs exposed to SARS-CoV-2, to describe the clinical presentation of COVID-19 in infected HCWs, and to investigate clinical and occupational risk factors for breakthrough infection. **Design:** Retrospective cohort study. **Methods:** The cohort of HCWs of Trieste Hospitals were followed up from 1 March 2020, to 30 November 2021 (21 months). All HCWs were periodically screened for SARS-CoV-2 infection by real-time PCR (RT–PCR) analysis. Clinical data were obtained through routine medical surveillance records. Risk factors for SARS-CoV-2 infection were investigated by univariable as well as multivariable logistic regression analysis. **Results:** Among 4394 HCWs routinely screened for SARS-CoV-2 by PCR on nasopharyngeal swab, a total of 800 incident cases were identified during the entire study period (1 March 2020 to 30 November 2021). Five hundred and sixty-four cases occurred before, and 236 after the start of the vaccination campaign against COVID-19, of whom 155 received a complete vaccination scheme before SARS-CoV-2 infection. Breakthrough infection was featured by mild or no symptoms and was significantly associated with the male sex, BMI > 25, and diabetes mellitus. Some categories of HCWs (physicians and nurse aids/auxiliary personnel) were at a higher risk of breakthrough infection. **Conclusions:** Fully vaccinated HCWs were less likely to acquire symptomatic as well as asymptomatic SARS-CoV-2 infection. Risk factors for SARS-CoV-2 infection after a full COVID-19 vaccination scheme included the male gender, diabetes mellitus, and overweight. HCWs with higher exposure to COVID-19 patients were at higher risk of breakthrough infection.

## 1. Background

A series of pneumonia of unknown origin in Wuhan, Hubei province, central China, was first reported to the World Health Organization (WHO) on 31 December 2019. The cause of the disease was identified in a new type of beta-coronavirus, the type 2 Severe Acute Respiratory Syndrome Coronavirus (SARS-CoV-2) responsible for coronavirus disease 2019 (COVID-19), a respiratory condition with potential to evolve into a severe pattern featured by a 3.4% mortality risk [1].

Since the declaration of the pandemic by the WHO on 11 March 2020, COVID-19 has affected hundreds of millions of people worldwide, thus far [2].

SARS-CoV-2 infects people via respiratory aerosol or direct contact with droplets during close face-to-face contact with infected people. Such characteristics make SARS-CoV-2 particularly dangerous for healthcare workers (HCWs), who are intrinsically at risk of occupational exposure to biological agents. Older adults, people with pre-existent co-morbidities, and HCWs, are reportedly at the highest risk for COVID-19 and its complications [3].

The first health protection measures enforced to contain the spread of COVID-19 were social distancing (including distance learning at school), hand washing, use of facemasks, and country-wide lock downs, imposed from 9 March to 3 May 2020. A mass vaccination against SARS-CoV-2 was undertaken with the aim to contrast the infection and alleviate the burden of the disease on healthcare systems [4,5].

For this purpose, four vaccines obtained authorization by the European Medicine Agency (EMA), i.e., Comirnaty (BNT162b2) manufactured by Pfizer–BioNTech, Spikevax (mRNA-1273), manufactured by Moderna Biotech, Vaxzevira (ChAdOx1), manufactured by AstraZeneca, and COVID-19 Vaccine Janssen (Ad26.COV2-S), manufactured by Johnson & Johnson [6].

In order to contrast the spread of SARS-CoV-2 pandemic, Italy and several other countries around the world started vaccination campaigns on a voluntary basis in December 2020, using the BNT162b2 messenger RNA vaccine (Pfizer–BioNTech) prioritizing HCWs and patients with chronic conditions.

The vaccination schedule for HCWs entails two-doses of BNT162b2 (30 μg per dose, given 21 days apart), a lipid nanoparticle-formulated, nucleoside-modified RNA (modRNA) encoding the SARS-CoV-2 full-length spike protein, that was found to be safe and 95% effective against COVID-19 [7].

HCWs receiving the BNT162b2 vaccine reportedly have a significantly lower incidence rate of symptomatic and asymptomatic SARS-CoV-2 infection, as compared to the unvaccinated, from 7+ days after the second dose [8].

The emergence of SARS-CoV-2 variants is seemingly compromising vaccine efficacy (VE), with post-vaccination infections outbreaks being reported in several countries worldwide [9,10,11,12,13,14].

Additionally, time-since-vaccination and decline in antibody levels seem to play a central role in breakthrough infections [15].

In this context, identifying risk factors in highly exposed populations becomes particularly important to prevent post-vaccination cases and outbreaks of COVID-19. 

In this study, we describe the impact of COVID-19 vaccination, the risk of breakthrough infection and the respective clinical course in a population of 4394 HCWs pertaining to two teaching hospitals affiliated to the University Health Agency Giuliano-Isontina (ASUGI) of Trieste (North-eastern Italy). 

## 2. Methods

### 2.1. Study Population

We conducted a retrospective cohort study designed to estimate the impact of the vaccination campaign, the course of SARS-CoV-2 infection among HCWs, and risk factors of breakthrough infection. 

The study included 4394 subjects working at ASUGI hospitals in Trieste and other healthcare facilities of the Giuliano area, North-eastern Italy. All of them were exposed to SARS-CoV-2 and were at risk of COVID-19; for this reason, an anti-COVID-19 vaccine was offered to all personnel, including administrative and logistic employees, according to national and hospital protocol for the COVID-19 emergency. 

During the period of the pandemic, in accordance with the protocol of the ASUGI pandemic plan, all personnel were periodically screened for SARS-CoV-2 infection through quantitative real-time reverse transcription–polymerase chain reaction (qRT–PCR) with specimen collected by a naso-pharyngeal swab (NPS) for the detection of viral RNA. HCWs underwent routine NPS based on the exposure risk to COVID-19 according to their job task, i.e., weekly, every other week, or monthly. Additionally, HCWs developing symptoms compatible with COVID-19 were also tested for SARS-CoV-2 infection, regardless of their routine screening schedule.

HCWs were included in the study if they met the following criteria:

- Being vaccinated with BNT162b2, vaccine (Pfizer Inc., New York, NY, USA; BioNTech SE, Mainz, Germany), Spikevax (mRNA-1273), manufactured by Moderna Biotech, Vaxzevira (ChAdOx1), manufactured by AstraZeneca or COVID-19 Vaccine Janssen (Ad26.COV2-S), manufactured by Johnson & Johnson;
- Having been tested for SARS-CoV-2 by RT–PCR on NPS specimen.

All infected HCWs were contacted by phone by the local infection prevention and control (IPC) unit of the local public health department, and invited to answer a survey tool, investigating demographic information, symptom severity, and any contact with confirmed cases of COVID-19.

In addition, date of vaccination and SARS-CoV-2 infection, information on age, sex, job task, work seniority, body mass index (BMI), smoking status and co-morbidities of the study subjects was collected for the analysis. Smoking was defined as current smoker vs. never or ex-smoker.

For HCWs eligible for the study, data about clinical conditions, occupation and smoking status were collected from medical surveillance records. 

The total incident cases of COVID-19 infection were divided into two groups:

- Incident cases between 1 March 2020 and 31 January 2021 (before the vaccination campaign);
- Incident cases between 1 February 2021 and 30 November 2021 (after the vaccination campaign).

The latter group was further divided into two sub-groups:

- A first sub-group including incident cases of fully vaccinated subjects;
- A second sub-group of fully unvaccinated or partially vaccinated subjects.

SARS-CoV-2 breakthrough infections among fully vaccinated HCWs were estimated among susceptible individuals (hence without history of COVID-19) testing positive 7+ days after the second dose of vaccine.

Information on age, sex, job task, work seniority, BMI, smoking status and co-morbidities of the reference population were obtained from a medical surveillance database. Cases with missing data were excluded and complete analysis was performed.

### 2.2. Biochemical Analysis

Viral RNA was extracted from naso-oropharyngeal specimens and analysed by RT–PCR targeting the E, N and RdRp genes of SARS-CoV-2 according to the CDC and Charité laboratory protocols. The cycle threshold (CT) values of RT–PCR were used as qualitative indicators of SARS-CoV-2 RNA viral load in specimens, with lower CT values corresponding to a higher viral load.

### 2.3. Statistical Analysis

Quantitative variables were expressed by mean (M) ± standard deviation (SD), while categorical variables by numbers and percentages.

Differences between continuous variables were assessed by Student’s *t*-test, and categorical variables with a chi-square test.

To investigate the respective risk factors, vaccinated HCWs with SARS-CoV-2 breakthrough infection were compared with vaccinated HCWs in the two Trieste hospitals without breakthrough infection. 

Factors associated to breakthrough infection were investigated using a univariable logistic regression analysis, selecting significant factors to be included in a multivariable logistic regression model. A *p*-value < 0.05 was considered statistically significant. Results were expressed as unadjusted, as well as an adjusted odds ratio (OR), with a 95% confidence interval (95%CI).

It must be noted that 14 incident cases were excluded from the analysis for missing data about vaccine date and clinical parameters, or because infection occurred < 7 days after second dose.

The study was conducted in accordance with the guidelines of the Good Clinical Practice and the ethical principles of Helsinki declaration; data were collected and archived in compliance with current legislation for observational studies using routine healthcare data. All data were treated anonymously. The local ethical committee (CEUR) of the Friuli-Venezia Giulia (FVG) region approved the study (Prot. 052/2021, 14 October 2021).

Missing data were excluded, and a complete case analysis was adopted.

STATA v16.0 (StataCorp LCC, Lakeway Drive, TX, USA) was employed for the analysis.

## 3. Results

From 1 March 2020 to 30 November 2021, 4394 HCWs of the two ASUGI hospitals in Trieste were routinely tested with RT–PCR for SARS-CoV-2 infection. Table 1 reports the distribution of the study population sex, age and COVID-19 vaccination status. As can be seen, 3003 (68.3%) were females and 1391 (31.7%) males. Population mean age was 46.8 ± 11.2 years, 46.9 ± 11.2 years for females, 46.7 ± 11.2 years for males.

After the detection of the first COVID-19 case on 9 March 2020, an overall number of 800 incident cases occurred. Before the COVID-19 vaccine campaign—from 1 March 2020 to 31 January 2021—564 (12.8%) of 4394 subjects tested positive for COVID-19 with an RT–PCR test among 4394 susceptible subjects, with an incidence rate of 128.4 cases per 1000 susceptible HVWs over 11 months.

The COVID-19 vaccination campaign started on 27 December 2020, and 1606 (36.5%) HCWs completed the respective vaccination schedule during the first month.

In total, 98.1% (=3558/3627) HCWs were vaccinated with BNT162b2 (Pfizer Inc., New York, NY, USA; BioNTech SE, Mainz, Germany), 1.8% (=65/3627) with Spikevax (mRNA-1273, by Moderna Biotech) and 4 with COVID-19 Vaccine Janssen (Ad26.COV2-S, by Johnson & Johnson) (Table 1).

From 1 February 2021 to 30 November 2021, 236 SARS-CoV-2 infections were detected among 3814 total susceptible HCWs (a rate of 6.9%), 4.9% (=81/657) among fully unvaccinated or partially vaccinated individuals and 12.3% (=155/3157) in vaccinated individuals.

Eight hundred incident cases of SARS-CoV-2 infections occurred from 1 March 2020) up to 30 November 2021, 67.7% (=542/800) among female HCWs. The mean age of incident cases of SARS-CoV-2 infection during this period was 44.7 ± 11.2 years, with identical distribution before (44.7 ± 11.4 years) and after (44.7 ± 10.7 years) 31 January 2021. After the start of the vaccination campaign, the difference in mean age between vaccinated (43.4 ± 10.8 years) and unvaccinated (47.2 ± 10.3 years) cases was statistically significant (*p* = 0.005) (Table 2).

Unvaccinated cases had significantly (*p* = 0.001) higher work seniority (12.4 ± 7.6 years) than the vaccinated cases (9.2 ± 7.8 years) (Table 2).

Among 800 total incident cases of SARS-CoV-2 infection during the overall study period (1 March 2020 up to 30/11/2021), 85% (=627/800) were symptomatic and 15% (=173/800) asymptomatic. Before the vaccine campaign the proportion of symptomatic SARS-CoV-2 infections was 94.5% (=494/564), from 1 February onward the latter ratio decreased to 62.1% (N = 133/236), 59.9% (=82/155) among vaccinated versus 66.3% (=52/81) among unvaccinated or partially vaccinated workers.

Symptomatic SARS-CoV-2 infections were featured by upper and lower respiratory tract symptoms, dyspnea, fever, cough, dysgeusia/anosmia, gastrointestinal symptoms, diarrhea, vomiting and other general systemic symptoms. The proportion of symptomatic infection declined for every disease manifestation after SARS-CoV-2 vaccination (with the exception of a cough), and between vaccinated vs. unvaccinated subjects (with the exception of lower respiratory tract symptoms).

One hundred and forty-five subjects became infected after a full vaccination schedule, of whom 139 (95.8%) were vaccinated with BNT162b2 (Pfizer Inc., New York, NY, USA; BioNTech SE, Mainz, Germany) and 2 with Spikevax (mRNA-1273).

As can be seen from Table 3, breakthrough infections were more frequent among nurse aids and auxiliary personnel (39%), followed by nurses (35.5%) and physicians (13.5%). The proportion of SARS-CoV-2 breakthrough infections among technicians (7.0%), and administrative staff (5.05) was considerably lower.

Table 4 shows the results of univariable as well as multivariable logistic regression analysis for the risk of SARS-CoV-2 breakthrough infection. As can be seen, males (OR = 1.59; 95%CI: 1.09; 2.29), individuals with BMI > 25 (OR = 1.57; 95%CI: 1.11; 2.23), and those affected by diabetes mellitus (OR = 2.41; 95%CI: 1.08; 5.37), were more likely to incur breakthrough infection at the univariate analysis. By contrast, the risk was significantly lower with linear increase in age (OR = 0.96; 95%CI: 0.95; 0.98) and for technicians (OR = 0.32; 95%CI: 0.14; 0.70) and administrative/support staff (OR = 0.34; 95%CI: 0.14; 0.84). Multivariable analysis confirmed the increased risk for males (OR = 1.61; 1.08–2.39), BMI > 25 (OR = 1.57; 1.07; 2.28), and diabetes mellitus (OR = 2.41; 1.04; 5.60). Older workers (OR = 9.95; 95%CI: 0.94; 0.97), technicians (OR = 0.29; 95%CI: 0.13–0.67) and administrative staff (OR = 0.43; 95%CI: 0.17–1.08) were again protected against infection.

## 4. Discussion

### 4.1. Key Findings

From 1 March 2020 through 30 November 2021 18.2% (=800/4394) HCWs of the two Trieste teaching hospitals were infected with SARS-CoV-2.

We counted 564 incident cases of SARS-CoV-2 infection over the course of 11 months before the start of the COVID-19 vaccination campaign and 236 infections in the following 10 months. The proportion of infected cases among susceptible HCWs was 12.8% (=564/4394) before the COVID-19 vaccination campaign (from 1 March 2020 up to 31 January 2021) and 6.9% (=236/3814) afterwards (1 February 2021 up to 30 November 2021).

After the start of the campaign SARS-CoV-2 infection rate was 4.9% (=155/3157) among vaccinated versus 12.3% (=81/657) in unvaccinated HCWs.

Among vaccinated HCWs the risk of breakthrough SARS-CoV-2 infection was significantly higher in males, individuals affected by diabetes and those with BMI > 25. By contrast, younger HCWs, technicians and administrative personnel were less likely to develop SARS-CoV-2 infection.

As regards with job task, the highest incidence of SARS-CoV-2 infection was found (by decreasing order of magnitude) among nurse aids/auxiliary personnel (39.0%), followed by nurses (35.5%), medical staff (13.5%) and technicians (7.9%), while administrative/support staff had the lowest rate (5.0%). Among vaccinated HWCs, technicians and administrative staff had lower risk of infection as compared with doctors at multivariable analysis, whereas nurse aids/auxiliary personnel had higher risk. However, the risk estimates were significant only for technicians.

### 4.2. Interpretation of Findings

#### 4.2.1. Incidence of SARS-CoV-2 Infection

As reported by several other studies, SARS-CoV-2 infection in HCWs followed the pandemic trend in the general population of the same geographical area [12,16].

In Italy, the vaccination campaign against COVID-19 started on 27 December 2020. Older adults, fragile patients and HCWs were identified as high-risk groups to be prioritized in the national vaccination campaign.

Thirty days after the start of the vaccination campaign, the infection rate of SARS-CoV-2 among HCWs in the two teaching hospitals of Trieste plummeted, confirming a VE reported also by a number of studies [4,5,7,8,17,18,19,20,21,22,23].

Although the efficacy of active immunization against COVID-19 to prevent the severe form of COVID-19 and death was reportedly nearly 100% in the respective clinical trials [7,24], shortly after the implementation of the vaccinations there was evidence that SARS-CoV-2 was able to colonize the upper airways of fully vaccinated HCWs, in some cases also propagating the infection to distant tissues, causing the typical clinical symptoms of COVID-19 [11]. In particular, emerging new variants of SARS-CoV-2 worldwide were reportedly capable to infect fully vaccinated individuals, causing symptomatic COVID-19 among HCWs. Booster immunizations may improve the humoral immune response against the Omicron variant, accuontable for a rapid increase of SARS-CoV-2 infections worldwide [25]. In our study we analyzed the risk of breakthrough SARS-CoV-2 infection among HCWs before booster immunization.

There is evidence that SARS-CoV-2 breakthrough infections are featured by significantly reduced viral loads in the nasal cavity at PCR analysis, thus their potential to shed the virus in the surrounding environment is likely limited [26]. According to our experience, symptomatic infections before the COVID-19 vaccine campaign occurred among 94.5% infected HCWs, whereas after 31 January 2021 the rate of symptomatic COVID-19 cases decreased to 62.1%.

#### 4.2.2. Risk Factors for Vaccine Breakthrough SARS-CoV-2 Infection

Little is known about the conditions predisposing to vaccine breakthrough SARS-CoV-2 infections in HCWs. In our study, male sex, younger age, BMI > 25, diabetes and job tasks were significant risk factors for breakthrough SARS-CoV-2 infection.

Whilst advanced age is associated with the highest risk of severe COVID-19 [27], younger individuals typically have increased inter-personal biological exposure due to higher frequency and intensity of social interactions. Furthermore, in Trieste teaching hospitals younger HCWs were predominantly assigned to COVID-19 wards since the beginning of the pandemic.

Despite in a matched case-control study on 164 vaccinated HCWs at Hamad Medical Corporation in Qatar between 20 December 2020 and 18 May 2021 breakthrough SARS-CoV-2 infection was not associated with co-morbidities >14 days after the second vaccine dose [28], males and individuals with chronic conditions are more likely to be infected by SARS-CoV-2 and to develop COVID-19 complications since the early days of the pandemic [27,29,30].

In fact, in a multi-centric Israeli study on 152 hospitalized patients developing COVID-19 > 7 days after the second dose of Pfizer/BioNTech’s BNT162b2 older age, co-morbidities (diabetes mellitus, arterial hypertension, heart failure, chronic, lung disease, chronic kidney disease and cancer) and immunosuppression were identified as potential risk factors for severe disease among fully vaccinated individuals [31]. Likewise, in a matched case-control study on USA veterans (502,780 vaccinated vs. 599,974 unvaccinated) diagnosed with SARS-CoV-2 infection from 15 December 2020 to 30 January 2021, during a follow-up time of 69,083 person-days in each group 2332 (0.5%) infections were recorded in vaccinated individuals > 14 days after the second dose of COVID-19 vaccine, against 40,540 (6.8%) among unvaccinated [32]. The latter US study also reported increasing risk of severe COVID-19 with age and among veterans with >4 comorbidities, but reduced in fully vaccinated veterans with breakthrough infection compared with unvaccinated controls infected by SARS-CoV-2 [32].

Albeit the risk of vaccine breakthrough SARS-CoV-2 infections in relation to spike-antibody levels after COVID-19 vaccination still has to be clarified, there is evidence that a decline of antibody levels over time may enhance this risk [33]. In particular, low titers of neutralizing antibody and S-specific IgG antibody have been proposed as predictors of SARS-CoV-2 breakthrough infection [9].

Three weeks after the first SARS-CoV-2 vaccine dose, IgG titres were reportedly lower in males, individuals aged 66+ years and immuno-depressed patients in 4026 serum samples from 2607 vaccinated HCWs followed up weekly for 5 weeks after the first vaccine dose in a single centre longitudinal cohort study at Sheba Medical Centre (Tel-Hashomer, Israel) from 19 December 2020 up to 30 January 2021 [34]. These differences were attenuated following the second vaccine dose (at week 3). Nonetheless, lower IgG levels were consistently associated with male sex, 66+ years of age, immuno-suppression, diabetes, hypertension, cardio-vascular disease and auto-immune disorders in the latter study [34]. Likewise, a recent review identified metabolic imbalance and diabetes associated with increased risk of vaccine breakthrough SARS-CoV-2 infection [27]. The evidence on decreasing IgG concentrations over time for males, older and immune depressed patients was also confirmed in a 6-month longitudinal prospective Israeli study on 4868 vaccinated HCWs testing monthly for the presence of anti-spike IgG and neutralizing antibodies [35]. Beyond hypertension and central obesity, lower antibody anti-spike titer were associated also with smoking in a single-centre study on 86 HCWs from a hospital in Rome (Italy) [36].

Further risk factors for vaccine breakthrough SARS-CoV-2 infection reportedly include anaemia, prior lung infection, chronic obstructive pulmonary diseases (COPD) and Alzheimer’s disease, whereas Black race seemingly decreases the risk [19,37].

As already reported by others [38], occupation was associated with risk of vaccine SARS-CoV-2 breakthrough infection in the present study. In particular, the highest incidence was found among nurse aids/auxiliary personnel, followed by nurses, doctors and technicians, whereas administrative/support staff had the lowest figures. All in all these results suggest a higher risk of infection in relation to job tasks entailing higher patient contact, as endorsed also by a recent systematic review [39]. However, seropositivity among HCWs may also be influenced by household contacts [30,31,32,33,34,35,36,37,38], an information which was not available in the present study though.

In order to prevent hospitals outbreaks among HCWs and patients, risk factors of breakthrough infections should be taken into consideration. For instance, the employment of HCWs affected by chronic conditions in COVID-19 designated wards might be restricted. Moreover, an appropriate screening strategy for high risk HCWs and their household members should be considered [40].

### 4.3. Strenghts and Limitations

The strengths of this work include the size of the study population, with rather good data completeness on vaccination status and symptoms, and the systematic weekly/monthly screening performed in HCWs to detect SARS-CoV-2 infection by molecular analysis on NPS specimen.

A possible weakness regards the relatively limited number of explanatory factors available for the analysis. Furthermore, we did not have information on SARS-CoV-2 variants involved.

Lastly, despite an almost identical length of time (11 vs. 10 months, respectively), the period before the start of the vaccination campaign included December and January, whereas in the period after the campaign the latter two winter months, featured by high circulation and transmissibility of SARSC-CoV-2, were excluded. This discrepancy may partly account for the difference in incidence between the two study periods (12.8% vs. 6.9%).

## 5. Conclusions

From 1 March 2020 through 30 November 2021 18.2% (=800/4394) HCWs of Trieste Teaching hospitals were infected with SARS-CoV-2, 12.8% (=564/4394) before versus 6.9% (=236/3814) after the start of the respective vaccination campaign. Fully vaccinated HCWs were less likely to develop asymptomatic as well as symptomatic SARS-CoV-2 infection as compared with their non-immunized colleagues. 

Males, individuals with BMI > 30 and those affected by diabetes mellitus were more likely to be infected by SARS-CoV-2 after a complete vaccination scheme. Furthermore, job tasks with higher level of exposure to COVID-19 patients (nurses aids/auxiliary personnel, nurses and doctors) increased the risk of SARS-CoV-2 infection.

The above findings should be taken into account by health care policies aimed at containing the risk of transmission of SARS-CoV-2 in health care settings.

Further research is recommended to monitor the antibody titre against of HCWs over time and the risk of breakthrough infection in relation to any future dose of COVID-19 vaccination, considering the impact of different SARS-CoV-2 variants and a higher number of clinical, behavioural and socio-demographic factors.

## Figures and Tables

**Table 1:** Distribution of study population by age, sex of heath care workers, vaccination status and type of COVID-19 vaccine administered. Number (N), row percentage (%).

FACTORS		VACCINATED (N = 3627)			UNVACCINATED (N = 767)		TOTAL (N = 4394)	
		Total	Females	Males	Males	Females	Males	Females
**No. (%)**		3627 (82.5)	3003 (68.3)	1391 (31.7)	546 (71.2)	221 (28.8)	3003 (68.3)	1391 (31.7)
**Age (years)** **M ± SD**		46.8 ± 11.2	46.9 ± 11.2	46.7 ± 11.2	47.7 ± 10.6	48.3 ± 11.0	46.9 ± 11.2	46.7 ± 11.2
**Type of COVID-19 Vaccine**	**BNT162b2** (Pfizer–BioNTech)	3558 (98.1)	2399 (97.9)	1158 (98.4)			2399 (79.9)	1158 (83.2)
	**mRNA-1273** (Moderna Biotech)	65 (1.8)	48 (1.9)	18 (1.5)			48 (1.6)	18 (1.3)
	**Janssen** (Johnson & Johnson)	4 (0.1)	3 (0.1)	1 (0.1)			3 (0.1)	1 (0.1)

**Table 2:** Distribution of incident SARS-CoV-2 infections among healthcare workers by explanatory factors, before and after the COVID-19 vaccination campaign. Number (N), percentage (%).

FACTORS		INCIDENT CASES N (%)				
		BEFORE THE CAMPAIGN (1 March 2020 to 31 January 2021) (N = 564)	AFTER THE CAMPAIGN (1 February 2021 to 30 November 2021) (N = 236)			Total (N = 800)
			Unvaccinated (N = 81)	Vaccinated (N = 155)	All	
**Cases/susceptible workers (%)**		564/4394 (12.8)	81/657 (12.3)	155/3157 (4.9)	236/3814 (6.9)	
**Sex**	**Males**	188 (33.3)	18 (22.2)	52 (33.6)	70 (29.7)	258 (32.3)
	**Female**	376 (66.7)	63 (77.8)	103 (66.4)	166 (70.3)	542 (67.7)
**Age (years)** **mean ± SD**		44.7 ± 11.4	47.2 ± 10.3 *	43.4 ± 10.8 *	44.7 ± 10.7	44.7 ± 11.2
**Seniority of work** (years) **M ± SD**		8.84 ± 7.6	12.4 ± 7.6 *	9.2 ± 7.8 *	10.33 ± 7.8	9.2 ± 8.7
**JOB TASKS**						
**Physician**		77 (13.7)	2 (2.5)	20 (12.9)	22 (9.3)	114 (14.2)
**Nurses**		250 (44.3)	28 (34.6)	56 (36.1)	84 (35.6)	326 (40.7)
**Nurse aids**		177 (31.4)	38 (46.9)	60 (38.7)	88 (41.5)	268 (33.5)
**Technicians**		22 (3.8)	4 (4.9)	11 (7.1)	15 (6.4)	42 (5.3)
**Administrative staff**		38 (6.8)	9 (11.1)	8 (5.2)	17 (7.2)	50 (6.3)
**SYMPTOMS**						
**All Symptoms**		494 (94.5)	51 (66.2)	82 (59.9)	133 (62.1)	627 (85)
**Upper respiratory tract**		181 (34.6)	27 (35.1)	41 (29.9)	68 (31.8)	249 (33.8)
**Lower respiratory tract**		5 (1)	0	2 (1.5)	2 (1)	7 (1)
**Dyspnea**		36 (6.9)	6 (7.8)	4 (2.9)	10 (4.7)	46 (6.2)
**Fever**		248 (47.4)	36 (46.8)	52 (38)	88 (41.1)	336 (45.6)
**Cough**		170 (32.5)	29 (37.7)	43 (31.4)	72 (33.6)	242 (32.8)
**Dysgeusia/Anosmia**		97 (18.6)	13 (16.9)	16 (11.7)	29 (13.6)	126 (17.1)
**Gastrointestinal**		27 (5.2)	4 (5.2)	5 (3.7)	9 (4.2)	36 (4.9)
**Diarrhoea**		24 (4.6)	3 (3.9)	5 (3.7)	8 (3.7)	32 (4.3)
**Vomiting**		4 (0.8)	1 (1.3)	0	1 (0.5)	5 (1.3)
**General symptoms**		208 (39.8)	32 (41.6)	53 (38.7)	85 (39.7)	293 (39.7)

* *p*-value < 0.005.

**Table 3:** Distribution of vaccinated healthcare workers of Trieste hospitals by explanatory factors and SARS-CoV-2 breakthrough infection. Number (N), column percentage (%) and *p*-value of chi-square (for categorical terms) or *t*-test (for linear terms). Bold numbers serve to highlight statistically significant results.

FACTORS	STRATA	SARS-CoV-2 BREAKTHROUGH INFECTION N (%)		*p*-Value
		YES (N = 141)	NO (N = 1279)	
**Sex**	**Males**	48 (34)	314 (24.6)	**0.01**
	**Females**	93 (66)	965 (75.4)	
**Age (years)** **M ± SD**		**43.4 ± 10.6**	47.2 ± 9.5	**0.00**
**BMI**	≤**25**	70 (49.6)	778 (60.8)	**0.01**
	**>25**	**71 (50.4)**	501 (39.2)	
**Smoking habit**	**Never/ex-smoker**	118 (83.7)	1033 (80.5)	0.36
	**Smoker**	23 (16.3)	246 (19.5)	
**Arterial hypertension**	**No**	130 (92.2)	1133 (88.5)	0.19
	**Yes**	11 (7.8)	147 (11.5)	
**Diabetes mellitus**	**No**	133 (94.3)	1249 (97.6)	**0.03**
	**Yes**	**8 (5.7)**	30 (2.4)	
**Occupation**	**Physicians**	**19 (13.5)**	129 (10.1)	**0.00**
	**Nurses**	**50 (35.5)**	483 (38)	
	**Nurse aids**	**55 (39.0)**	281 (22.1)	
	**Technicians**	**10 (7.0)**	214 (18.8)	
	**Administrative staff**	**7 (5.0)**	139 (11)	

**Table 4:** Univariable and multivariable logistic regression analysis on the risk of SARS-CoV-2 breakthrough infection among vaccinated healthcare workers of Trieste hospitals. Odds ratio unadjusted (OR) and adjusted (aOR) with 95% confidence interval (CI) and stratum specific *p*-value. Significant estimates are reported in bold.

FACTORS	STRATA	UNIVARIABLE ANALYSIS		MULTIVARIABLE ANALYSIS	
		OR (95%CI)	*p*-Value	aOR (95%CI)	*p*-Value
**Sex**	**Female**	*reference*		*reference*	
	**Males**	**1.59 (1.09; 2.29)**	**0.01**	**1.61 (1.08; 2.39)**	**0.02**
**Age** (linear) (years)		**0.96 (0.95; 0.98)**	**0.00**	**0.95 (0.94; 0.97)**	**0.00**
**BMI** (Kg/m^2^)	**≤25**	*reference*		*reference*	
	**>25**	**1.57 (1.11; 2.23)**	**0.01**	**1.57 (1.07; 2.28)**	**0.02**
**Smoker**	**never/ex**	*reference*			
	**Current**	0.80 (0.50; 1.28)	0.35		
**Arterial hypertension**	**No**	*reference*			
	**Yes**	0.65 (0.34; 1.23)	0.17		
**Diabetes mellitus**	**No**	*reference*		*reference*	
	**Yes**	**2.41 (1.08; 5.37)**	**0.04**	**2.41 (1.04; 5.60)**	**0.04**
**Occupation**	**Physicians**	*reference*		*reference*	
	**Nurses**	0.70 (0.40; 1.23)	0.22	0.67 (0.38; 1.19)	0.17
	**Nurse aids/auxiliary personnel**	1.33 (0.76; 2.33)	0.32	1.40 (0.79; 2.49)	0.25
	**Technicians**	**0.32 (0.14; 0.70)**	**0.01**	**0.29 (0.13; 0.67)**	**0.00**
	**Administrative and support staff**	**0.34 (0.14; 0.84)**	**0.02**	0.43 (0.17; 1.08)	0.07

## Data Availability

The datasets generated and analysed during the current study are not publicly available, since they were purposively collected by the authors for the present study, but may be available from the corresponding author on reasonable request.
